# Severe Epistaxis in the Pediatric Patient: A Simulation for Emergency Department Management

**DOI:** 10.7759/cureus.27784

**Published:** 2022-08-08

**Authors:** Olivia Gorbatkin, Jean Pearce, Monique Goldschmidt, Anita Thomas, Elizabeth Sanseau, Daisy Ciener, Regina Toto, Ashley E Keilman

**Affiliations:** 1 Pediatrics, University of Washington, Seattle, USA; 2 Pediatrics, Medical College of Wisconsin, Milwaukee, USA; 3 Gastroenterology and Hepatology, Nationwide Children’s Hospital, Columbus, USA; 4 Pediatric Emergency Medicine, Seattle Children’s Hospital, Seattle, USA; 5 Pediatric Emergency Medicine, Children’s Hospital of Philadelphia, Philadelphia, USA; 6 Pediatric Emergency Medicine, Vanderbilt University Medical Center, Nashville, USA

**Keywords:** volume resuscitation, nasal packing, pediatric emergency, simulation, liver disease, hemorrhagic shock, epistaxis

## Abstract

Severe, uncontrolled epistaxis in a pediatric patient can lead to a compromised bloody airway and the potential need for significant volume resuscitation secondary to hemorrhagic shock if not managed emergently. In this report, a simulated 11-month-old patient with underlying liver disease presents to the emergency department setting. The goal was to familiarize advanced pediatric emergency medicine trainees and experienced providers with immediate bedside interventions and clinical management steps for a patient with severe, difficult-to-control epistaxis to increase preparedness for future clinical scenarios. Additionally, this case highlights resuscitation considerations for patients with liver disease, including sources of bleeding, consulting services, medications, and approach to massive transfusion in liver disease.

## Introduction

Epistaxis is common in the pediatric emergency department (ED) and can often improve with basic, non-emergent interventions, namely, direct compression [1]. Uncontrolled severe epistaxis can be a life-threatening medical emergency. It is especially rare in children under two years old (1 per 10,000) but warrants immediate consideration of underlying bleeding disorders, trauma, tumors, or esophageal sources such as varices [2]. Due to its relatively rare presentation, providers may be less familiar with early, effective interventions to prevent hemodynamic instability or airway compromise in a pediatric patient. Simulation of uncommon high-risk presentations can better prepare medical professionals to provide optimal treatment and protect patient safety while increasing provider skills and confidence [3].

The goal of this simulation case is to increase medical providers’ technical skills and clinical knowledge of effective emergent management for severe epistaxis in the pediatric population while considering underlying medical complexities that could complicate interventions and outcomes such as biliary atresia. These complications include sensitivity to volume overload in liver disease during resuscitations [4,5]. Additionally, this simulation will challenge providers to manage a bloody airway, considering specific supplies, alternative techniques, and assistance from difficult airway teams [6].

Specifically, ED providers should be familiar with early bedside interventions, including nasal packing and topical medications, early consultation with otolaryngology (ENT), and considerations for underlying liver diseases, especially in volume resuscitation. This simulation serves as an opportunity to practice institution-specific massive transfusion protocol and difficult airway supplies and teams. This simulation will focus on these objectives with emphasis on the critical actions necessary for prompt management of a complex scenario for intermediate to advanced learners familiar with standard Pediatric Advanced Life Support (PALS) algorithms seeking to build upon that foundational knowledge.

## Technical report

This simulated scenario occurs in the setting of an ED with standard resuscitation equipment (oxygen, suction, intubation supplies, medications, nasal packing, etc.) and consultants available by phone. The simulated scenario was conducted using a pediatric simulator and was led by a facilitator experienced with pediatric resuscitation. Team roles are consistent with institutional care team structure and may include team lead, bedside provider, bedside nurse, family communicator, consultant communicator, pharmacist, simulation technician, and observers. These optional roles could be performed by participants attending in person or virtually.

This simulation was developed and run with a total of 41 participants across four academic pediatric emergency departments. Participants included residents, pediatric emergency medicine (PEM) fellows, ENT physicians, and PEM attendings. Each location was provided with the simulation scenario, supplemental slides for post-simulation education, and a post-simulation participant survey. Physical examination findings were provided verbally by facilitators if unable to be ascertained by participants examining the manikin.

Below are the outlined learning objectives for the simulation case provided in this report.

1. Discuss the management approach for suspected anterior and/or posterior nosebleeds:

a. Specify bedside interventions (ideal positioning, nasal packing, topical medications).

b. Verbalize the need for early ENT consultation.

2. Verbalize the need for blood products:

a. List key lab tests for a patient presenting with hemorrhage.

b. Demonstrate the use of a massive transfusion protocol.

c. Describe the modified approach to massive transfusion given underlying liver disease.

3. Discuss the considerations and indications for intubation to protect the airway in case of nasopharyngeal or oral bleeding. Verbalize the need for airway backup given anticipated difficulty.

Pre-briefing

A facilitator overview summarizing participant roles, bedside supplies, learning objectives, and critical actions steps for the scenario is provided in Table 1. Didactic slides may be presented before or after scenario participation or asynchronously. With participants present, roles should be assigned. Prior to the simulation, participants should be oriented to the manikin, be made aware of the safe learning environment provided by simulation, and session expectations should be clearly outlined.

**Table 1:** Facilitator overview, pediatric emergency medicine simulation: severe epistaxis. ED = emergency department; PICU = pediatric intensive care unit; CBC = complete blood count; CMP = complete metabolic panel; PT/INR = prothrombin time/international normalized ratio; PTT = partial thromboplastin time

Patient: Tina, Age: 11 month old, Weight: 10 kg, Chief complaint: nosebleed	
Brief case description	Tina is an 11-month-old female with a history of end-stage liver disease secondary to biliary atresia presenting to the ED for epistaxis. On arrival at the ED, she is awake, alert, breathing comfortably, and tachycardic, but normotensive. Anticipated interventions include bedside treatment of epistaxis, early ENT consult, and recognition and management of progressive hemorrhagic shock. Despite these interventions, the patient will have worsening epistaxis and concern for airway compromise, requiring intubation. The team may discuss the need for fluid vs. blood resuscitation, and the team will be asked if they want to initiate a massive transfusion protocol (institution dependent) to treat hemorrhagic shock. Participants will need to re-evaluate the patient’s clinical stability and the effectiveness of their interventions. The case will conclude with intubation and transfer to the PICU
Participant roles (required)	Simulation facilitator, Team lead/head of the bed, Bedside provider
Participant roles (optional, virtual or in person)	Co-simulation facilitator, Bedside nurse, Family communicator, Consultant communicator, Pharmacist, Simulation technician, Critical action checklist reviewer, Observers
Supplies	Monitors, oxygen with mask interfaces, suction, intubation supplies, airway adjuncts (supraglottic airway, oropharyngeal airway, etc), medications, nasal packing sponge, nasal balloon catheter with syringe, institution-specific massive transfusion protocol algorithm
Learning objectives, 1	Discuss management approach for suspected anterior and/or posterior nosebleeds. A. Demonstrate bedside interventions (ideal positioning, nasal packing, topical medications). B. Verbalize the need for early ENT consultation
Learning objectives, 2	Verbalize the need for blood products. A. List key lab tests for the patient presenting with hemorrhage. B. Recognize the need for blood products, including modifications to massive transfusion protocol on underlying liver disease
Learning objectives, 3	Discuss considerations and indications for intubation to protect the airway in case of nasopharyngeal or oral bleeding. Verbalize the need for airway backup given the anticipated difficulty
Critical actions, Clinical state #1: Initial assessment	1. Examine the patient and place monitors. 2. Verbalize or perform bedside interventions. 3. Obtain labs/diagnostic studies (CBC, CMP, PT/INR, PTT, fibrinogen, type and screen, blood gas. Consider blood culture). 4. Collect focused history. 5. Verbalize differential diagnosis for epistaxis. 6. Consult ENT and hepatology (not available to come emergently or will arrive in 15 minutes)
Critical actions, Clinical State #2: Worsening bleeding involving the oropharynx	1. Identify worsening bleeding. 2. Attempt bedside nasal packing (if not gone in State #1). 3. Attempt topical hemostatic medication (if not gone in State #1). 4. Identify worsening vitals and the concern for hemorrhagic shock. 5. Initiate fluid resuscitation. 6. Initiate transfusion per institutional protocol (fill in): _____Packed red blood cells,_____Fresh frozen plasma, _____Platelets, _____Other
Critical actions, Clinical state #3: Airway management	1. Identify airway compromise due to blood in the oropharynx. 2. Perform endotracheal Intubation. 3. Confirm intubation with chest X-ray. 4. Adjunct airway as needed
Critical actions, Clinical state #4: Stabilization of patient, admission to PICU	Transfer to PICU
Ideal scenario flow	Once the patient is roomed, the team correctly identifies concern for epistaxis vs. esophageal variceal bleeding and applies packing/topical medications, obtains IV access, and sends labs. The team will initiate volume resuscitation and implement a massive transfusion protocol (per institution guidelines). The team recognizes the need to secure the airway and PICU transfer. Participants will need to continually re-evaluate the patient’s clinical stability and the effectiveness of their interventions
Anticipated management mistakes	1. Failure to optimize bedside hemostasis with appropriate positioning, pressure, and nasal packing with topical medication. 2. Failure to recognize the importance of early ENT consult. 3. Failure to consider esophageal varices as a potential bleeding source. 4. Delayed recognition and treatment of hemorrhagic shock via fluid resuscitation and massive transfusion in a patient with underlying liver disease. 5. Failure to recognize management of difficult airway - preparation, equipment, and consultant (anesthesia) resources

Case

**Figure 1:**
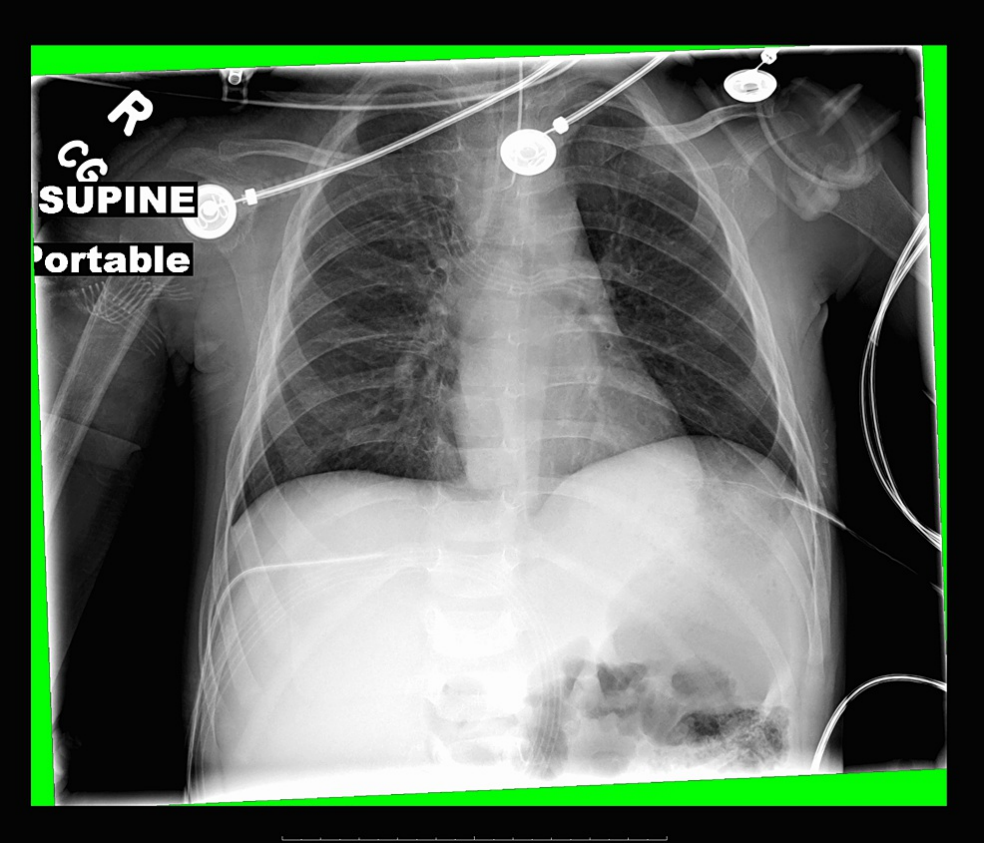
Portable chest X-ray.

This simulation case is an 11-month-old female with a history of end-stage liver disease secondary to biliary atresia presenting to the ED for greater than 20 minutes of epistaxis that cannot be controlled at home with direct compression. The case begins with the facilitator reading the triage note (Table 2) and proceeds with participants entering the emergency department room where the simulated patient is sitting on the bed connected to monitors. Once requested by participants, the history, vitals, and physical examination findings are provided by the facilitator (Table 2). The case will advance guided by the actions of the participants, and the facilitator can follow the stepwise progression of the case announcing changes along the way (Table 3). When requested by the participants, labs and imaging can be shown (Table 4, Figure 1).

**Table 2:** Initial presentation, State #1. HPI = history of present illness; PMH = past medical history; PERRL = pupils equal and reactive to light and accommodation; EOMI = extraocular muscles intact

Initial presentation	
Triage note: 1 liner	11-month-old, being held on parent’s lap with active bleeding from nares. Awake and alert
Triage note: Vitals	Heart rate 140, oxygen saturation (SpO_2_) 97%, blood pressure 90/60, respiratory rate 30, temperature 36.8°C, weight 10 kg
HPI	Tina is an 11-month-old female with end-stage liver disease secondary to biliary atresia presenting with uncontrolled epistaxis. She is brought in by her mother after a 25-minute nosebleed. The patient has had prior nosebleeds that have resolved at home in 10 minutes. No history of trauma. Mom reports subjective fever, attributed to teething. Denies other associated symptoms
HPI: Sample history (if asked)	Signs/symptoms: fever, nasal bleeding. Allergies: none. Meds: parent unable to recall. PMH: end-stage liver disease secondary to biliary atresia, currently awaiting a liver transplant. Unable to proceed with transplant 1 month ago due to viral illness, but has been stable since convalescence. Last intake: about 3 hours ago. Events preceding: as above
Additional history	Past medical/surgical history: biliary atresia. Medications: parents unsure. Allergies: none. Family history: none. Social history: lives with mom at home. No pets
Physical examination (primary assessment, secondary assessment)	
Vitals	Heart rate 170, oxygen saturation (SpO_2_) 97%, blood pressure 90/60, respiratory rate 44, temperature 36.8°C
General	Anxious, sitting on parent’s lap, leaning back with active nasal bleeding, more distressed with exam
HEENT	Patent airway, no signs of head trauma, PERRL, EOMI, (+) scleral icterus, no hemotympanum, bilateral nares obstructed by the blood which is becoming increasingly brisk, uncooperative and flailing when attempting nasal speculum exam, oropharynx initially clear though blood starts to appear in the posterior oropharynx
Neck	Supple
Lungs	Slightly tachypneic, clear to auscultation bilaterally, no stridor or wheezing
Cardiovascular	Tachycardic, 2+ peripheral pulses, capillary refill 2-3 seconds
Abdomen	Abdomen soft, nontender, mildly distended with liver edge felt ~2 cm below right costal margin, unable to palpate spleen, normal bowel sounds, no masses, no fluid wave or bulging flanks.
Neurological	Opening eyes spontaneously, spontaneous movements, making sounds and crying, pupils 3 mm to 2 mm, moving all extremities equally, no focal deficits
Skin	No rash or bruises. Few telangiectasias on the abdomen. Skin jaundiced
Genitourinary	Normal genitourinary examination
Psychiatric	Fearful of examiners, and appropriate clinging to caregiver

**Table 3:** Stepwise progression of care. PICU = pediatric intensive care unit

Stepwise progression of the case		
Intervention/Time Point in scenario	Change in case	Additional information
State #1, Initial presentation: Triage nurse notifies the team of patients arrival, providers go to the bedside	Learners should enter the patient room with assigned team roles, assess ABCs, confirm monitors in place, ask for current history and vitals, and examine the patient	See Table 2 for HPI, current vitals, and physical exam
Bedside management of epistaxis	The learner should optimize head positioning (forward tilt, nose down, and firmly compress nasal cartilage just inferior to nasal bone). Can perform oral suctioning. If correct positioning and compression occur, nasal bleeding slows but continues. The learner should recognize the need to call ENT emergently and discuss bedside nasal packing options (supplies including topical medication agents). Consider hepatology consult	Packing options: foley, nasal packing sponge, nasal balloon
Nasal packing initiated on patient	Learner should request material. If learner does not know what material to request, nurse can prompt with, “We have a bedside nasal balloon or a foley.” If the learner does not request a topical medication on packing material, a nurse can ask, “Would you like any topical medication to place on the packing materials to help with clotting?” The case advances to the next section after 1-2 minutes with continued oozing blood from the nose	Packing materials: nasal packing sponge, nasal balloon, foley, suction. Topical medications: oxymetazoline, phenylephrine TXA, lidocaine
IV access obtained x 1. Lab results requested. State #2 triggered when the patient begins vomiting bright red blood or after 5 minutes from case start	Lab results available upon request. If the participants do not request any labs, can have the embedded participant nurse prompt when putting in the IV: “Do you want me to collect any blood for labs?” Learners should contact hepatology specialists due to concern for esophageal varices, need for additional interventions	EPOC labs immediately available: pH: 7.37, pCO_2_: 37 mmHg, pO_2_: 43 mmHg, Na: 134 mmol/L, K: 4.4 mmol/L, Ca: 8.6 mg/dL, Cl: 105 mmol/L, glucose: 100 mg/dL, lactate: 1.9 mmol/L, creatinine: 0.2 mg/dL, hematocrit: 31 %. See Table 4 for additional labs available at a later time point
State #2, Worsening: Vitals reassessed. Heart rate 190, blood pressure 75/40, respiratory rate 50, SaO_2_ 96%, Temperature 37.0°C, ETCO_2_ patient unable to tolerate/obtain nasal ETCO_2_. Physical exam: as initial, but with bright red emesis, 4-5 sec CR and feeling cooler, seems sleepier, and vital signs as above	If the team doesn’t initiate fluid resuscitation, the nurse asks, “Are we worried about hemorrhagic shock?” The nurse asks, “She has significant blood loss and worsening tachycardia. When should we consider the massive transfusion protocol?” If the team does not ask for the second point of IV access, the nurse should prompt, “Do you want me to get another IV line on this patient?”	
Volume resuscitation	If >15 mL/kg blood is given or MTP initiated, advance to State 3 or after 10 minutes in State 2. Additionally, consider octreotide	If check bedside EPOC Hgb again, should be 6. If hepatology not yet consulted, prompt learner with, “Are there any additional consultants who you would like to call?” If no blood ordered after 3 minutes, facilitator can prompt with, “Are there any additional fluids or meds you want to give?” Note, octreotide is initiated as bolus of 1-2 µg/kg (max 100 µg), followed by 1-2 µg/kg/hour IV infusion, titrated to response (3-4 µg/kg/hour maximum)
State #3, Airway management: Once the airway is secured, move to State #4. Or if the airway not secured after 10 minutes in State #3, end scenario. Physical exam: same as State #3 (until sedated/paralyzed for airway)	The facilitator states there are copious bloody secretions in the oropharynx. The learner should identify pooling blood in the oropharynx without source control. If intubation is deferred, saturations decrease to the upper 80s from likely aspiration. If the team proceeds with intubation, note sedation meds, ETT size, and blade size. The team should discuss calling for anesthesia (or the most experienced advanced airway provider). If the team does not call, the facilitator to note and discuss in debrief	ETT: 3.5-4.0 cuffed. Blade: MIL 1 Intubation meds for sedation: etomidate 0.3 mg/kg, ketamine 1-2 mg/kg. Not as ideal due to cause of hypotension/unstable: propofol 1-1.5 mg/kg, midazolam 0.2-0.3 mg/kg. Other: fentanyl 1-5 µg/kg. Intubation meds for paralysis: rocuronium 1-1.2 mg/kg. If a chest X-ray requested post-intubation, it will show ETT at 1.7 cm above the carina. See Figure 1. ETC0_2_: 38 or positive color change
State #4. Stabilization: Completion and signout to PICU/25 minutes. Vitals: Heart rate 160, blood pressure 85/45, respiratory rate 40, SaO_2_ 98%, temperature 37.0°C. Physical exam: sedated/paralyzed, blood in bilateral nares, airway secured, tachycardic, perfusion 2-3 seconds, CTAB, abdomen continues with mild distension/telangiectasis, liver edge felt about 2 cm below RCM		The facilitator can play the role of PICU attending. Sample sign out: “11 mo F with liver failure presenting with hemorrhagic shock 2/2 to epistaxis vs variceal bleed, given ___ blood products, ___ fluid, airway secured with TT. Should consider bcx and abx given potential gastrointestinal bleed with risk of bacterial translocation not ruled out at this time.”

**Table 4:** Labs. pCO_2_ = venous carbon dioxide; pO_2_ = venous oxygen; Na = sodium; K = potassium; Ca = calcium; Cl = chloride; Glu = glucose; Lac = lactate; Crea = creatinine; Hct = hematocrit; WBC = white blood cells; Hbg = hemoglobin; Plt = platelets; CO_2_ = bicarbonate; BUN = blood urea nitrogen; Mg = magnesium; Phos = phosphorous; Total Bili = total bilirubin; Conj = conjugated bilirubin; Unconj = unconjugated bilirubin; AST = aspartate aminotransferase; ALT = alanine transaminase; GGT = gamma-glutamyl transferase; PT = prothrombin time; INR = international normalized ratio; APTT = activated partial thromboplastin clotting time; VBG = venous blood gas; Bicarb= bicarbonate; Temp = temperature

EPOC labs immediately available	Sent to lab
pH: 7.37	WBC: 8.3 K/µL
pCO_2_: 37 mmHg	Hbg: 10.5 g/dL
pO_2_: 43 mmHg	Hct: 31 %
Na: 134 mmol/L	Plt : 95 THOU/µL
K: 4.4 mmol/L	
Ca: 8.6 mg/dL	Na: 134 mmol/L
Cl: 105 mmol/L	K: 4.4 mmol/L
Glu: 100 mg/dL	Cl: 105 mmol/L
Lac: 1.9 mmol/L	CO_2_: 16 mmol/L
Crea: 0.2 mg/dL	BUN: 4 mg/dL
Hct: 31%	Creat: 0.2 mg/dL
	Glucose: 100 mg/dL
	Albumin: 2.8 g/dL
	Ca: 8.6 mg/dL
	Mg: 2.1 mg/dL
	Phos: 4.0 mg/dL
	Total Bili: 11.4 mg/dL
	Conj: 9.1 mg/dL
	Unconj: 2.3 mg/dL
	AST: 304 U/L
	ALT: 1331 U/L
	Lipase: 491 U/L
	GGT: 498 U/L
	PT: 19 seconds
	INR: 1.6
	APTT: 40 seconds
	Fibrinogen: 134 mg/dL
	Lactic acid: 1.9 mmol/L
	VBG
	pH: 7.37
	pCO_2_: 37 mmHg
	pO_2_: 43 mmHg
	Bicarb: 21 mmol/L
	Base excess: -3.8
	Temp: 38.6 °C
	Type and screen: B+

Debriefing

After the conclusion of the simulation case, participants were led through a facilitated debrief. Participants were encouraged to discuss successful and challenging portions of the scenarios, as well as review learning objectives. Facilitators, experienced simulation educators with expertise in debriefing methodology, utilized advocacy inquiry, plus-delta, and the PEARLS framework at their discretion based on participant performance and educational needs [7]. If participants conducted interventions that were not a part of the anticipated stepwise progression, the simulation facilitator reviewed ideal interventions during each state. Depending on the level of the learners, facilitators could consider discussion of evidence-based medicine, institutional practice variation, system resources, and other treatment considerations.

Post-scenario didactics

After the scenario, simulation facilitators reviewed the severe epistaxis didactic figures with emphasis on reinforcing the learning objectives. These figures review the differential for epistaxis, nasal anatomy and sites for bleeds, bedside interventions with visuals of supplies and placement, labs, massive transfusion protocol, considerations in volume resuscitation in liver failure, and bloody airway management. The figures are provided below with additional explanations addressing each objective.

Objective 1

Discuss management approach for suspected anterior and/or posterior nosebleeds:

a. Demonstrate bedside interventions (ideal positioning, nasal packing, topical medications).

b. Verbalize the need for early ENT consultation.

**Figure 2:**
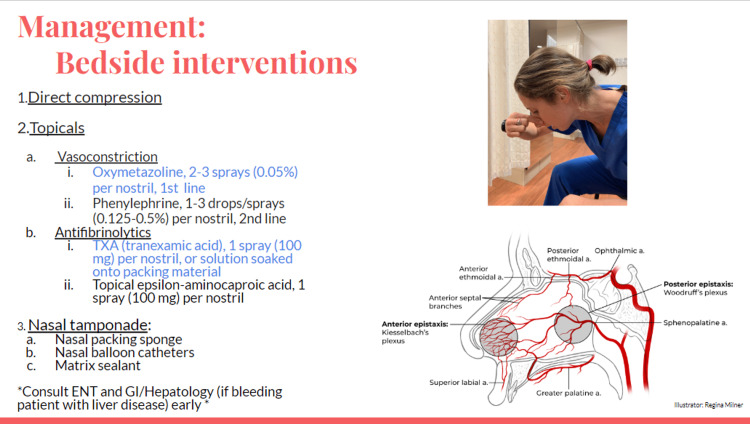
Bedside interventions slide.

**Figure 3:**
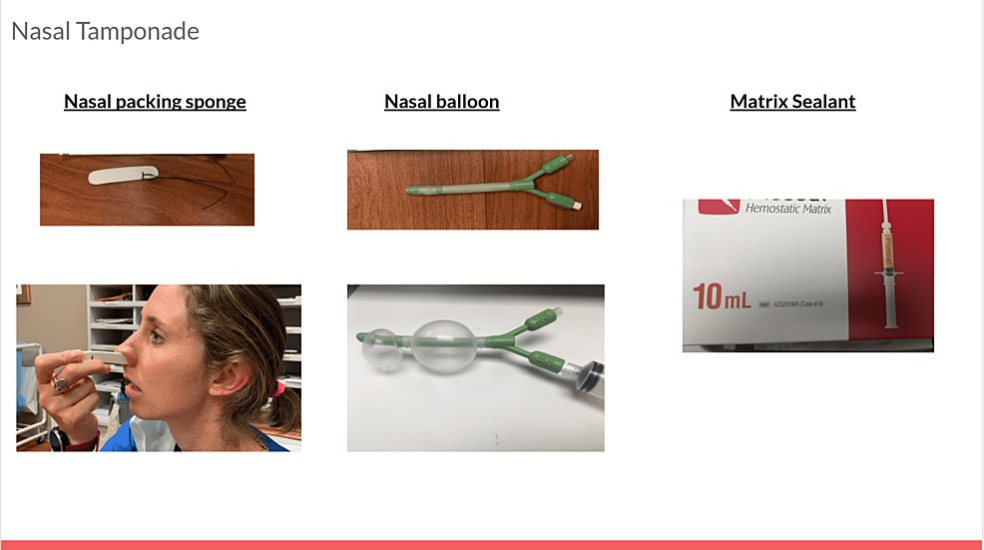
Nasal tamponade slide.

Anterior epistaxis is more common and originates in Kiesselbach’s plexus. Posterior epistaxis is less common and originates in Woodruff’s plexus. Direct pressure with the patient tilted forward decreases ingestion or aspiration of blood [1]. Second line is topical medications, aimed at vasoconstriction (oxymetazoline, phenylephrine) or antifibrinolytics (tranexamic acid, epsilon-aminocaproic acid), though there is varied data on efficacy. Oxymetazoline generally remains first line before tranexamic acid [8]. Third line is nasal packing (sponge or balloon), which is more likely to reach posterior sources than direct pressure. Packing is aimed slightly toward the nasal septum and occiput, avoiding going superiorly. For nasal packing, leave strings hanging out of the nose. For nasal balloon, inflate the balloons after placement with air to improve tamponade. Matrix sealant is an additional option. ENT should be consulted in accordance with institutional practice. For this patient with an unknown source of bleeding, participants should also consider early hepatology consultation to direct interventions, including octreotide infusion, antibiotic therapy, and possible endoscopic variceal ligation (Figures 2, 3).

Objective 2

Verbalize the need for blood products:

a. List key lab tests for a patient presenting with hemorrhage.

b. Demonstrate use of a massive transfusion protocol.

c. Describe the modified approach to massive transfusion given underlying liver disease.

Recognition and treatment of hemorrhagic shock is critical to stabilization and clinical outcomes. Besides point-of-care hematocrit, electrolytes, blood gas, and lactate should be obtained if available. Hypoperfusion should be considered with lactates greater than 2 mmol/L, and increased mortality with initial lactates greater than 4-5 mmol/L [9].

**Figure 4:**
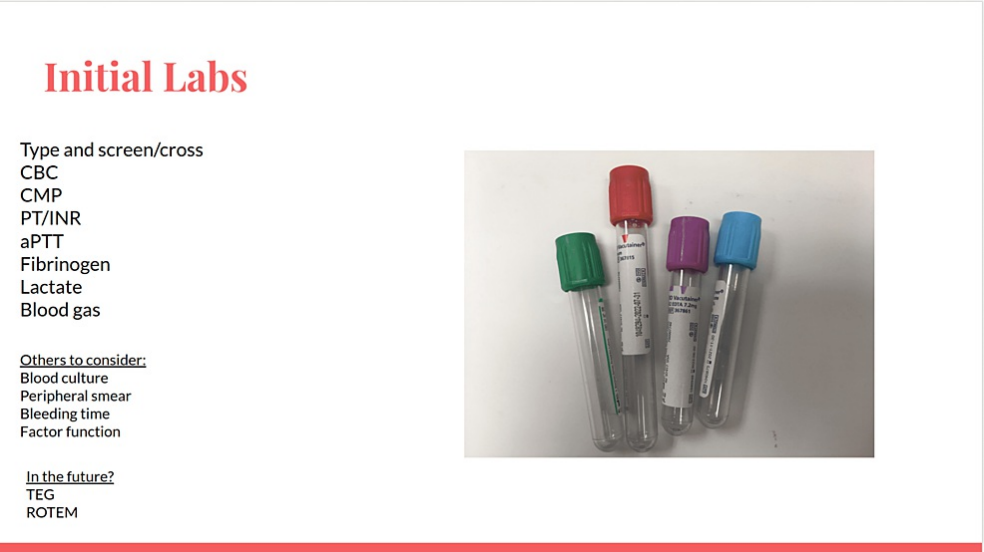
Initial labs slide.

**Figure 5:**
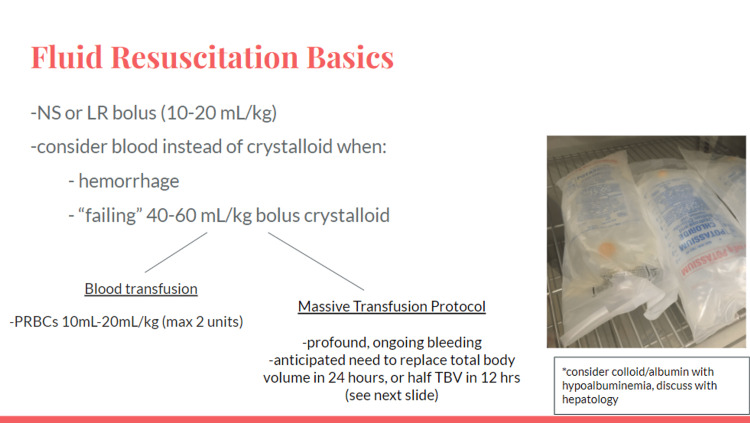
Volume resuscitation slide.

**Figure 6:**
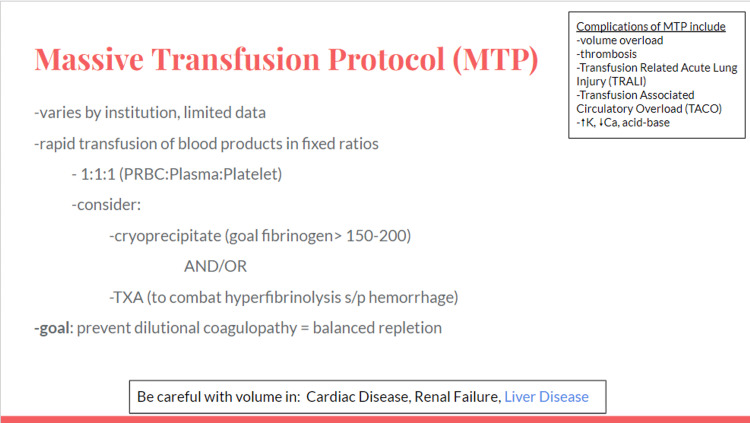
Massive transfusion protocol slide.

**Figure 7:**
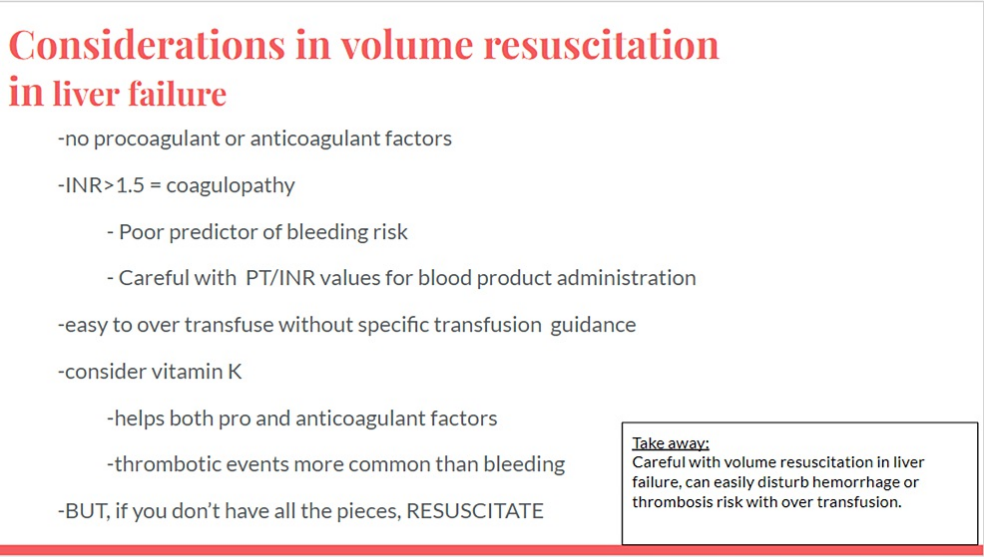
Considerations in volume resuscitation in liver failure slide.

Massive transfusion should be considered with profound bleeding. Facilitators discussed institution specific massive transfusion protocols. Volume resuscitation complications with massive transfusion protocol include volume overload leading to pulmonary edema (transfusion-associated circulatory overload), thrombosis, and transfusion-related acute lung injury. Liver disease with portal hypertension prompts cautious volume resuscitation similar to heart disease and renal failure [10]. If deliberate but cautious volume can be given safely (i.e., restrictive transfusion for goal 7-9 g/dL), it is favored to not increase intravascular pressure and exacerbate variceal bleeding [4]. Additional considerations in liver disease include hypoalbuminemia and hepatorenal syndrome (Figures 4-7).

Objective 3

Discuss considerations and indications for intubation to protect the airway in case of nasopharyngeal or oral bleeding. Verbalize the need for airway backup given anticipated difficulty.

**Figure 8:**
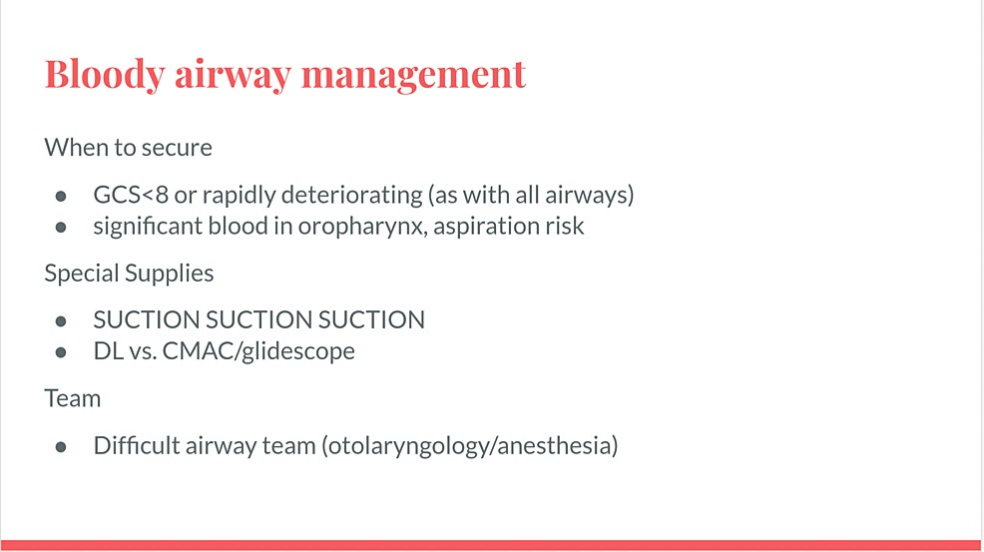
Airway management slide.

Intubation in a pediatric patient could be necessary for airway protection from blood in the oropharynx, significant aspiration, and/or due to clinical deterioration in the setting of continued hemorrhage. Facilitators emphasized familiarity with advanced intubation equipment including video laryngoscopy and teams that can aid with a difficult airway (anesthesia or ENT) [6]. Bedside suction will be essential during intubation. The simulation facilitator is encouraged to review the use of these supplies with participants (Figure 8).

Following scenario participation, learners completed a simulation survey. Results are summarized in Table 5.

**Table 5:** Simulation participant survey results. Median response from Likert scale: 1 = strongly disagree; 2 = disagree; 3 = neutral; 4 = agree; 5 = strongly agree.

Question	Median (n)	Range
How likely are you to recommend this session to a colleague?	5 (41)	3-5
Following the simulation, I feel comfortable with bedside management of epistaxis including nasal compression, topical medications, and nasal tamponade	4 (41)	3-5
Following the simulation, I understand the need to consult otolaryngology early in uncontrolled epistaxis	5 (30)	4-5
Following the simulation, I have a better understanding of when and how to initiate the massive transfusion protocol	5 (41)	2-5
Following the simulation, I understand how to approach continued bleeding in the setting of underlying liver disease and the need for thoughtful fluid resuscitation	5 (35)	4-5
Following this simulation, I feel comfortable managing a bloody oropharynx, including types of suction, intubation equipment, and when to activate a difficult airway team	4 (30)	4-5
Following this simulation, I have a better understanding of when to consider intubation to protect the airway in case of nasopharyngeal or oral bleeding if the Glasgow Coma Scale score is normal	4 (11)	3-5
The debrief promoted reflection and team discussion	5 (41)	4-5

## Discussion

Severe epistaxis in a pediatric patient is a low-frequency but high-risk presenting condition in the ED that can lead to hemorrhagic shock and airway compromise. Gaining experience with bedside interventions, differential diagnosis, and management decisions is important for the clinical knowledge base of trainees working in the pediatric ED. This simulation aims at familiarizing providers with key bedside interventions with the goal of strengthening technical skills and critical stabilization interventions that must be initiated prior to subspecialty consultant arrival. Specifically, participants practice initial bedside management with direct compression, nasal packing, and topical vasocontraction or antifibrinolytic-directed medications while involving early consultation (ENT, hepatology, difficult airways team). Access is critical, and two vascular access sites should be obtained. These early interventions are critical for initial site control. In the case of underlying liver disease, variceal bleeding should be considered and treated with octreotide when a source cannot be confirmed for a patient in hemorrhagic shock [11]. Participants are familiarized with institution-specific massive transfusion protocol and reasons for initiating. Volume resuscitation in a patient with underlying liver disease should be approached in a similarly cautious manner to underlying renal disease or heart failure. Emergency medicine providers should strongly consider the urgent involvement of hepatology specialists per institutional practice given the nuances in management and the need to deviate from standard massive transfusion protocols.

Feedback from each of the four sites and 41 participants was utilized to iteratively revise the scenario and didactic materials. The participant survey was also modified in parallel to correspond to case objectives given varied total responses. The participant survey was modified mid-way through this project to include the learning objective regarding the protection of the airway; 11 participants responded to the revised question, as reflected in Table 5. The post-simulation participant survey showed provider comfort level with technical skills and clinical knowledge in our stated learning objectives. Learners reported an increased understanding of key resuscitation objectives after simulation participation, with a median response of 4-5 on a Likert 1-5 scale (Table 5). Based on this post-survey response, we believe this simulation was an effective educational session to achieve improved preparation for managing severe epistaxis.

Given institutional variability, this simulation aims to have flexibility in its design to fit local educational needs including the number and training level of participants, in-person or virtual presence for certain participant roles, type of simulators available, presence of consultants, and background knowledge of facilitators. Some high-fidelity mannequins may not permit nasal packing. Facilitators could consider using a task trainer, allowing participants to verbalize completion of nasal packing, subspecialty consultation, or show a brief educational video demonstrating the correct technique during the debrief. The didactic materials were used to clarify and reiterate key learning concepts. Facilitators may incorporate the didactic materials prior to the simulation scenario to increase baseline clinical knowledge or follow the debrief to reinforce the learning objectives based on the training level of participants.

## Conclusions

Management of severe epistaxis in pediatric patients is an uncommon but high-risk condition requiring emergent intervention with the potential for progressive airway compromise and hemorrhagic shock. This multi-center simulation was effective for teaching key interventions and clinical concepts for uncontrolled epistaxis based on participant and facilitator feedback. This scenario allows trainees to practice rapid assessment skills, consider the differential diagnosis, administer bedside interventions, and increase familiarity with system resources. Importantly, it may be tailored to institutional treatment protocols. Knowledge of when to deviate from standard treatment algorithms and tailor resuscitation efforts to the individual patient, in this case modifying fluid resuscitation and massive transfusion for hemorrhagic shock in a pediatric patient with underlying liver disease, is a key learning point for advanced PEM trainees.
